# Bleeding risk in female patients undergoing intravesical injection of onabotulinumtoxinA for overactive bladder: a Danish retrospective cohort study

**DOI:** 10.1007/s00192-023-05579-1

**Published:** 2023-06-17

**Authors:** Meryam El Issaoui, Sophia Elissaoui, Marlene Elmelund, Niels Klarskov

**Affiliations:** 1 Department of Gynecology and Obstetrics, Herlev and Gentofte University Hospital, Borgmester Ib Juuls Vej 1, 16. Etage, 2730 Herlev, Denmark; 2 Department of Clinical Medicine, University of Copenhagen, Copenhagen, Denmark

**Keywords:** Urgency urinary incontinence, OnabotulinumtoxinA injections, Antithrombotic therapy, Antiplatelets, Anticoagulants, Bleeding, Hematuria

## Abstract

**Introduction and hypothesis:**

We aimed to examine the risk of bleeding in female patients undergoing intravesical onabotulinumtoxinA (BTX-A) treatments and provide clinical recommendations for the perioperative management of patients on antithrombotic therapy prior to BTX-A treatments.

**Methods:**

This was a retrospective cohort of Danish female patients, who had their first BTX-A treatment because of an overactive bladder at the Department of Gynecology and Obstetrics, Herlev and Gentofte University Hospital, between January 2015 and December 2020. Data extraction was from an electronic medical journal system. BTX-A, Botox® Allergan was injected in the detrusor at 10–20 sites. Significant bleeding during or after a BTX-A treatment was defined as persistent macroscopic hematuria. Bleeding reporting was based on information obtained from journal notes.

**Results:**

We included 400 female patients, who had a total of 1,059 BTX-A treatments. Median age at first BTX-A treatment was 70 years (IQR 21), and median number of BTX-A treatments was 2 (range 1–11). In total, 27.8% (*n*=111) received antithrombotic therapy. Within this group, 30.6% and 69.4% were on anticoagulant and antiplatelet therapy. No cases of hematuria were reported in our cohort. We found that no patients stopped their antithrombotic therapy, were bridged, or monitored by International Normalized Ration (INR) levels.

**Conclusions:**

We suggest that BTX-A treatments might be classified as low-risk procedures. Discontinuation of antithrombotic therapy is not required in the perioperative management of this patient group.

## Introduction

Intradetrusor onabotulinumtoxinA (BTX-A) injections are a well-established treatment for overactive bladder. The injections are performed intravesically with a needle in 10–20 sites in the detrusor under cystoscopic guidance [1]. More than 600 BTX-A treatments are performed yearly at our tertiary Urogynecology Clinic, Herlev and Gentofte University Hospital, and the number is increasing.

It is an invasive procedure that can potentially result in bleeding complications. The risk of perioperative bleeding, in general, is increased in patients of older age (>65 years), patients with cardiovascular and cerebrovascular diseases, abnormal renal or liver function, and on certain medicines [2, 3]. Risk assessment of bleeding complications involves an overall assessment of patient-related and procedure-related risks for thrombosis and bleeding [4]. Female patients referred for BTX-A treatments tend to be older, and older age is associated with cardiovascular and cerebrovascular diseases [5, 6] requiring antithrombotic therapy. The clinical management of antithrombotic therapy for patients who require an invasive procedure is a common challenge. To our knowledge, there are no guidelines on the management of patients on antithrombotic therapy undergoing BTX-A treatments. Cystoscopy is listed as a low-risk procedure, but other transurethral procedures, such as bladder biopsy, are listed as moderate-risk procedures, in which discontinuation of antithrombotic therapy is indicated [7]. In addition, bridging may be indicated depending on the antithrombotic agent [4]. However, the Danish Association on Thrombosis and Hemostasis, which conducts guidelines regarding bridging for surgical procedures, has not listed BTX-A injections in the currently available bridging application commonly used by Danish physicians [8]. No clear consensus exists on the approach to the perioperative management of antithrombotic therapy for patients undergoing BTX-A treatment, and updated recommendations are needed. Our study was aimed at examining the risk of bleeding in female patients undergoing intravesical BTX-A treatments and to provide clinical recommendations for the perioperative management of antithrombotic therapy. We hypothesized that the risk of bleeding requiring intervention might be low, and that discontinuation of antithrombotic therapy was not required.

## Materials and methods

Approval of our retrospective cohort study was obtained from Capital Region’s Knowledge Center for Data reviews (ID: R-21013125). Data extraction of our cohort of female patients, who had their first BTX-A treatment at the Department of Gynecology and Obstetrics, Herlev and Gentofte University Hospital between January 2015 and December 2020, was from the Danish electronic medical journal system “Sundhedsplatformen” by a data specialist. Patient data including demographics, medical history, antithrombotic therapy and indication, number of BTX-A treatments, information on bridging, International Normalized Ratio (INR) monitoring, and bleeding reporting were reviewed. All collected data were stored in the online database Research Electronic Data Capture (REDCap).

### Study population

Female patients over 18 years of age referred for treatment with BTX-A injections owing to an overactive bladder in our Urogynecology Clinic were identified.

### BTX-A treatment

Patients were treated with BTX-A, Botox® Allergan. BTX-A was injected into the detrusor at 10–20 sites sparing the trigone under cystoscopic guidance. The treatments were performed in an operating theater or at our outpatient clinic, depending on the requirement for sedation during the injections.

### Bleeding reporting

Significant bleeding during or after a BTX-A treatment was defined as persistent macroscopic hematuria. Easy bleeding from the injection sites may occur but usually ceases during the BTX-A injection procedure, causing no significant hematuria.

Patients undergoing BTX-A treatments are routinely informed to contact our outpatient clinic if they experience any adverse effects, including bleeding. Follow-up related to assessment of post-void residual volume is scheduled for some patients, whereas no specific follow-up of bleeding is scheduled routinely. Bleeding reporting was based on information obtained from journal notes in the medical records.

Patients on antithrombotic therapy were divided in two groups depending on the administered antithrombotic agent, anticoagulant (AC), and antiplatelet (AP) therapy respectively.

### Statistical analysis

Descriptive statistics were performed in R Studio, version 2022.07.1. Age and number of treatments were reported in median, interquartile range (IQR), and range. In this analysis, we assessed rates of antithrombotic therapy, antithrombotic agents, and the primary outcome, bleeding.

## Results

We included 400 consecutive female patients who had intravesical BTX-A treatments. Baseline characteristics are listed in Table 1. In total, 1,059 BTX-A treatments were performed during the study period. Median age at first BTX-A treatment was 70 years (IQR 21, range 19–92). The patients received between 1 and 11 BTX-A treatments with a median of 2 treatments (range 1–11). BTX-A treatment doses varied between 50 IU and 300 IU. In total, 27.8% (*n*=111) of patients received antithrombotic therapy during their first BTX-A treatment. Seven different antithrombotic agents were registered, as listed in Table 2. Within this group, 69.4% and 30.6% were on AP and AC therapy respectively. One patient (0.9%) administered dual AP therapy with aspirin and clopidogrel. Antithrombotic therapy indications included previous stroke, cardiovascular diseases, and prevention of thromboembolism. We found no reported cases of bleeding or hematuria. No patients were discontinued in their antithrombotic therapy, bridged, or monitored by INR levels.

**Table 1:** Baseline characteristics

Characteristic	Data
Total number of patients, *n*	400
Age at first BTX-A treatment^a^	70 (56–77)
Total number of BTX-A treatments	1,059
Number of BTX-A treatments^b^	2 (1–11)
Antithrombotic therapy indication	
Cardiovascular diseases^c^	82 (73.9)
Previous strokes^c^	27 (24.3)
Thromboembolic diseases^c^	2 (1.8)

BTX-A Onabotulinum toxin A

^a^Median (q1–q3)

^b^Median (range)

^c^Total number (percentage, %)

**Table 2:** Overview antithrombotic therapy, *n* (%)

Characteristic	Data
Number of patients on antithrombotic therapy^a^	111 (27.8%)
Anticoagulant therapy	34 (30.6%)
Vitamin K antagonists	
Warfarin	2 (1.8%)
Direct thrombin inhibitors	
Pradaxa	2 (1.8%)
Direct factor Xa inhibitors	
Apixaban	23 (20.7%)
Rivaroxaban	7 (6.3%)
Antiplatelet therapy	77 (69.4%)
Aspirin	36 (32.4%)
Clopidogrel	40 (36.0%)
Dual therapy with aspirin and clopidogrel	1 (0.9%)

^a^Total number (% of total population number) at the first otulinumtoxinA treatment

## Discussion

We found no cases of bleeding in a Danish retrospective cohort comprising 400 female patients, who underwent between 1 and 11 BTX-A treatments for an overactive bladder at our Urogynecology Clinic. Within this cohort, 27.8% of patients were on antithrombotic therapy. Our results demonstrate that BTX-A treatments constitute a low bleeding risk. A previous Cochrane review concluded that intravesical BTX-A treatments are safe, with few adverse effects and complications, thereby supporting our results [1]. Three of the studies included in the review each reported one case of hematuria in small populations [9–11]. One study reported a single subject experience of hematuria after placebo injection with saline, which after further investigation seemed to originate from ureteric varicosities [10]. Another study reported hematuria in a patient who received a suburothelial BTX-A injection [11]. Mensah et al. recently reported a hemorrhagic event in one male patient, requiring a 1-day treatment of a two-way urethral catheter, out of 114 patients on antithrombotic therapy in a cohort comprising 532 female and male patients. The patient was on nonvitamin K oral anticoagulant (NOAC) therapy with rivaroxaban and received BTX-A treatment with 300 U at 20 sites. Additional patient-related risk factors included a history of prostate radiotherapy and self-catheterization [12]. Our cohort is unique in only including female patients, as male patients have been reported to have hematuria rates between 1.3% and 29% [13].

We found that none of the patients in our cohort discontinued their antithrombotic therapy prior to BTX-A treatments, according to the medical record. A plausible explanation could be that the procedure in our department is considered safe from a clinical perspective. However, antithrombotic therapy challenges the perioperative management of this patient group, and international consensus on this clinical matter is lacking. Older patients on antithrombotic therapy are at a higher risk of thromboembolic events and complications, including bleeding [3], and discontinuation of antithrombotic therapy perioperatively could further contribute to these perioperative events [14, 15]. Previous studies reported an association between AC discontinuation and perioperative thromboembolic events, with reported rates up to 1.9% [14, 15]. In a study, recurrent stroke and cardiovascular event rates following discontinuation of the AP agents, aspirin plus extended-release dipyridamole (ASA + ERDP) or clopidogrel, were compared with event rates in an on-treatment population. The reported absolute excess risks of recurrent stroke within 30 days of discontinuation were 0.77% for ASA + ERDP and 0.40% for clopidogrel. The corresponding absolute risks of major vascular events were 1.83% and 2.02%. AP discontinuation after ischemic stroke should be advocated when the risk of bleeding outweighs the risk of cardiovascular events [16]. Therefore, a balanced risk assessment of the patient and procedure-related risk of bleeding and thromboembolism is important in managing antithrombotic therapy for patients referred for an invasive procedure. The European Society of Cardiology (ESC) has recently updated a guideline on the cardiovascular assessment and management of patients undergoing noncardiac surgery, including perioperative management of antithrombotic agents [4]. Patient risks include age, individual thrombotic risk, history of bleeding complications, renal function, concomitant medication, and comorbidity, whereas procedure-related risk factors include urgency of the intervention and the bleeding risks of the procedure [4]. The perioperative management of antithrombotic therapy depends on the administered antithrombotic agent. Seven different antithrombotic agents were administered in the cohort, of which 30.6% of patients were administered AC agents during their first BTX-A treatment. Generally, AC therapy discontinuation is not recommended prior to low-risk bleeding procedures and other procedures with easy bleeding control [17]. Low-risk bleeding procedures are recommended to be performed at trough levels (12 to 24 h after last intake of AC), scheduling the procedure at the start of the next dosing interval, in patients on NOAC therapy (direct thrombin inhibitor and direct factor Xa inhibitors) [4, 18]. Monitoring and maintenance of the INR at the lower level of the therapeutic range is recommended, particularly in patients with mechanical heart valves and on vitamin K antagonists [1, 17]. Conversely, guidelines on the perioperative management of patients undergoing urological surgery recommend discontinuation of the vitamin K antagonist warfarin 3–5 days prior to the procedure for patients who are at a low risk for thromboembolism [19].

Among patients on antithrombotic therapy, we found that 69.4% were administered AP agents. The ESC recommends that continuation of the AP agent aspirin should depend on the perioperative bleeding risk compared with the risk of thrombotic complications [17]. Guidelines on perioperative AP management in patients undergoing minor procedures are lacking [20]. A previous randomized trial reported no significant difference in the risk of a major thromboembolic event and bleeding, whether low-dose aspirin was continued or discontinued in 291 patients with cardiac risk factors who underwent noncardiac surgery. The trial included 45 unspecified urological major surgeries [21]. The American Urological Association recommends continuation of low-dose aspirin in patients undergoing prostatic biopsies, based on a slightly increased bleeding risk [22]. This is supported by a recent meta-analysis, that reported no increased risk of hematuria after a prostate biopsy. However, a risk of postoperative rectal bleeding was reported [23]. Transurethral resections of bladder tumors have been reported to be safe concerning bleeding, as a previous retrospective cohort study found no increased risk of severe bleeding. However, an increased risk of postoperative clot retention was reported. Results were based on a small female and male population, including 19 on antithrombotic therapy, 18 discontinuing antithrombotic therapy, and 137 in the control group [24]. We can argue that BTX-A treatments are less invasive than a prostate biopsy procedure and transurethral resection of bladder tumors, emphasizing that BTX-A treatments may be considered a low-risk procedure.

For the prevention of a perioperative thromboembolism during discontinuation of AC therapy, a bridging therapy strategy can be used. Bridging therapy refers to the use of short-acting ACs, including unfractionated heparin or low-molecular-weight heparin [25]. We found that none of the patients in our cohort was bridged. Bridging benefits in patients are open to debate because of an increased risk for stroke owing to AC discontinuation, whereas bridging may cause a higher bleeding risk without necessarily protecting against thromboembolism [22, 26, 27]. On the contrary, international urological guidelines recommend bridging therapy before surgery or when the INR is lower than 2.0 in patients on warfarin therapy [19]. INR monitoring was not performed in our patient cohort. INR monitoring is essential for patients on warfarin therapy, whereas monitoring INR in patients on NOACs is not required owing to different pharmacokinetics [28].

The bleeding risk associated with BTX-A treatments is not well described, and to our knowledge no guideline is available on the management of patients on antithrombotic therapy undergoing this procedure, emphasizing the need for recommendations.

Based on our findings and the available literature, we suggest that BTX-A treatments might be classified as low-risk surgical procedures with regard to bleeding complications. As the benefits of antithrombotic therapy are likely to outweigh the risk of bleeding, demonstrated by no reported bleeding case in our cohort, we suggest continuing antithrombotic therapy in female patients undergoing a BTX-A treatment. BTX-A treatments are frequently performed at our Urogynecology Clinic, and the number is increasing. Our results can support the development of a clinical practice guideline concerning the perioperative management of antithrombotic therapy, including counseling, in this patient group. Nevertheless, physicians should assess patients individually in the case of a history of procedure-related bleeding.

In the event of bleeding, treatment is accessible. Common treatments of hematuria in urogynecological patients include urethral catheterization with a two-way catheter and saline bladder irrigation. Bleeding management at the operating theatre could be indicated in the case of failed conservative treatment, or a hemodynamically unstable patient.

Our study has some limitations. We may have underestimated the bleeding risk and missed some cases of bleeding, as any events relied on self-reporting from the patients. Patients are informed to contact the Urogynecology Clinic in the event of bleeding. No systematic reporting or follow-up on bleeding has been established. The retrospective nature of the study contributes to limitations as well. Inconsistent reporting in the medical record by medical staff could also contribute to missing data on bleeding cases. Selection bias was avoided by including 400 consecutive female patients in the study period.

## Conclusion

We have demonstrated that the risk of hematuria requiring treatment after BTX-A injections is low in female patients on antithrombotic therapy. Thereby, BTX-A treatments are a low-risk surgical procedure, and we suggest that antithrombotic therapy should not be discontinued during the perioperative management of this patient group.
